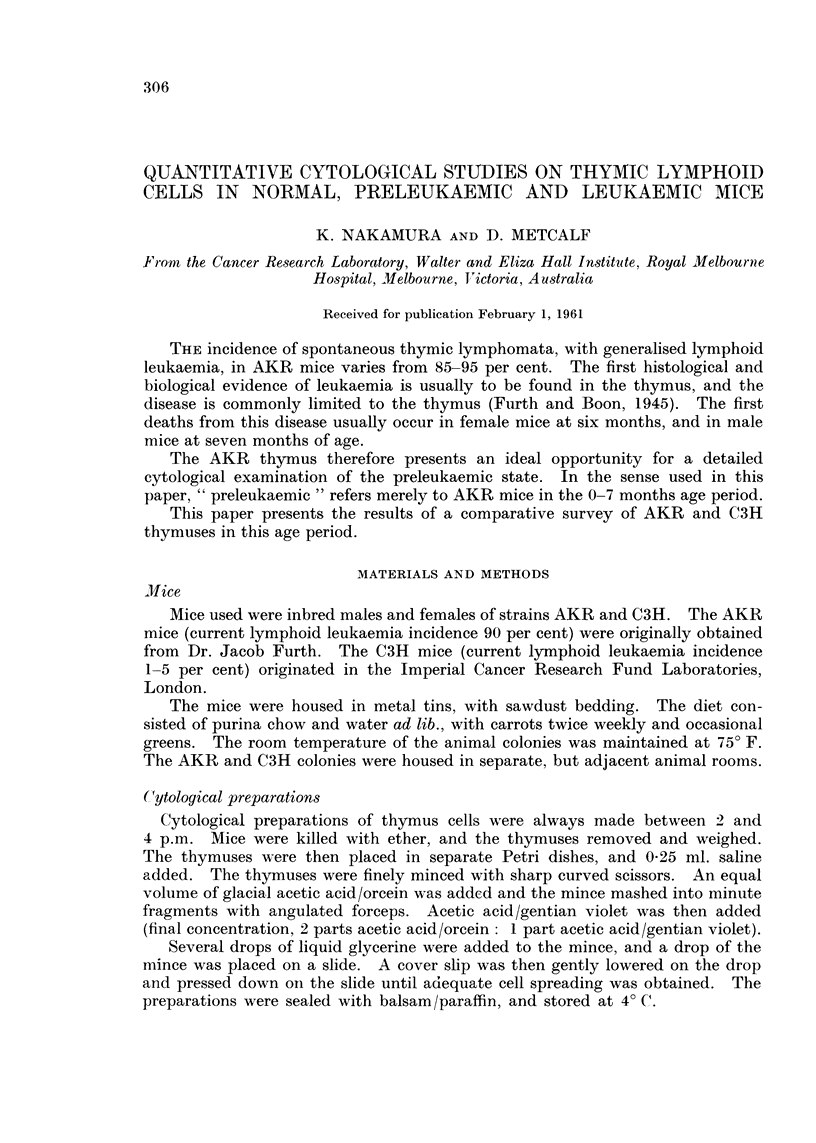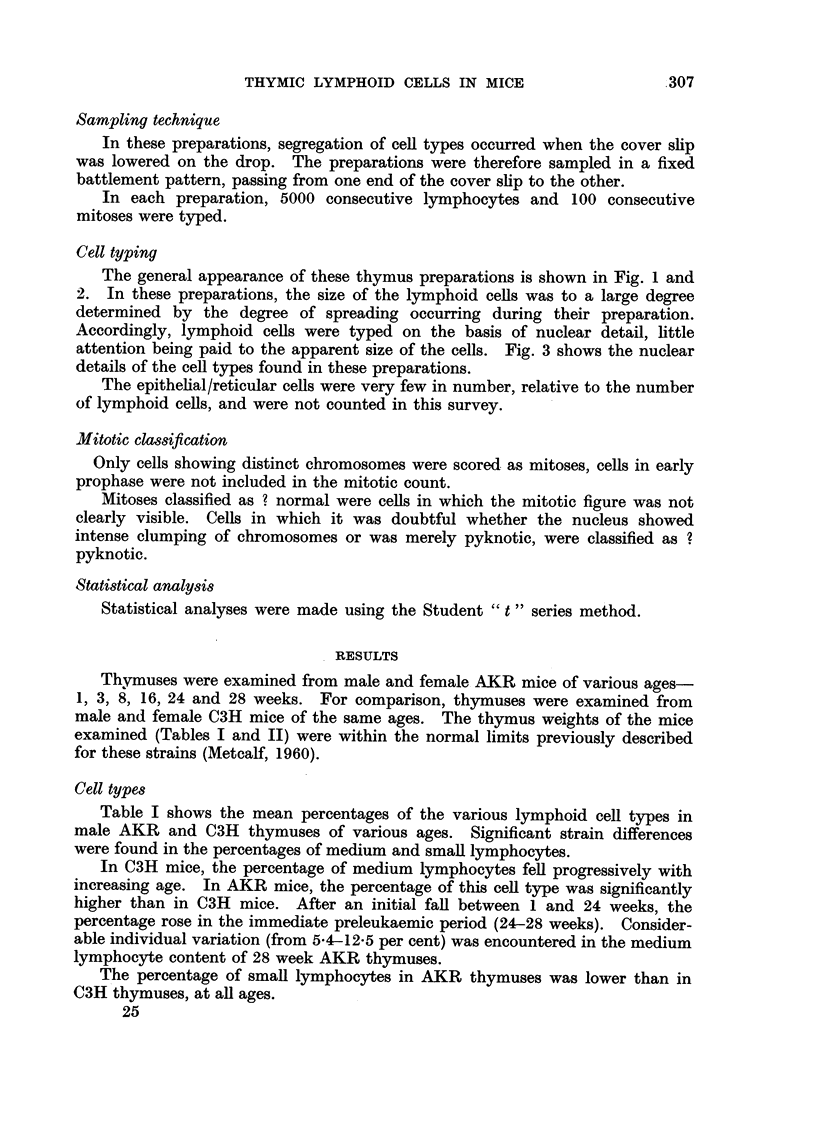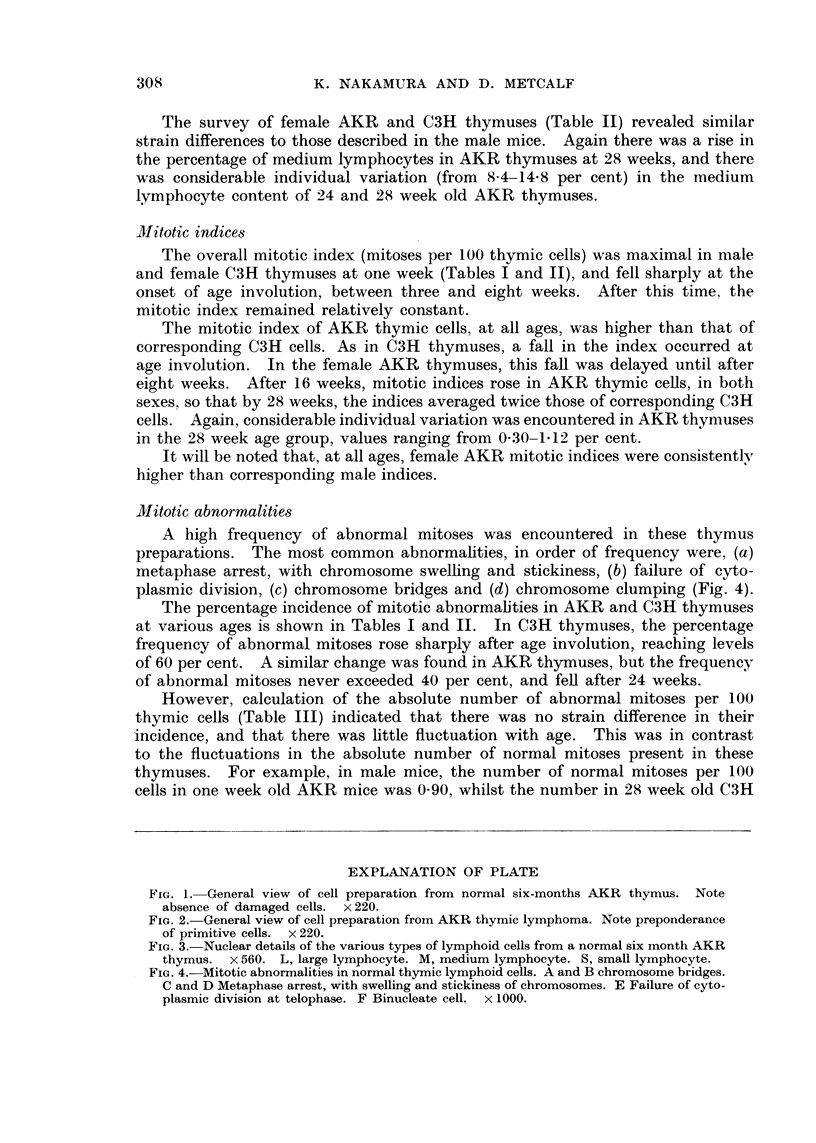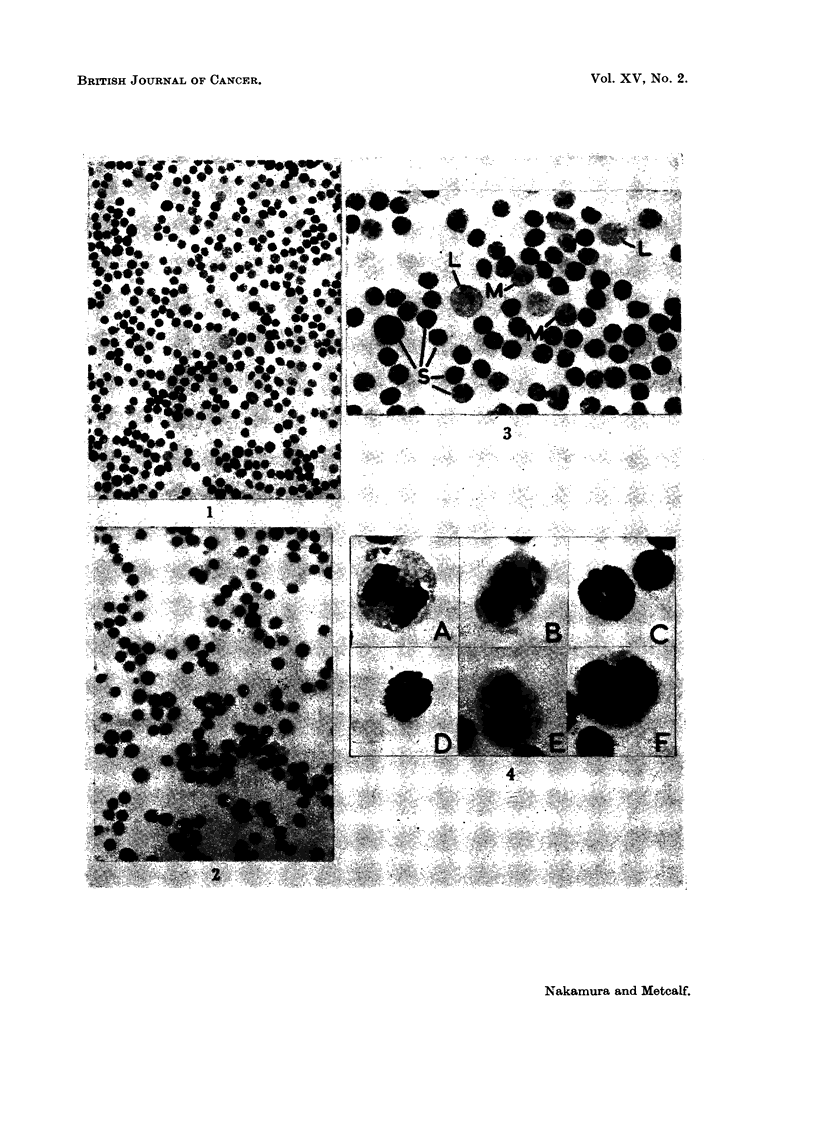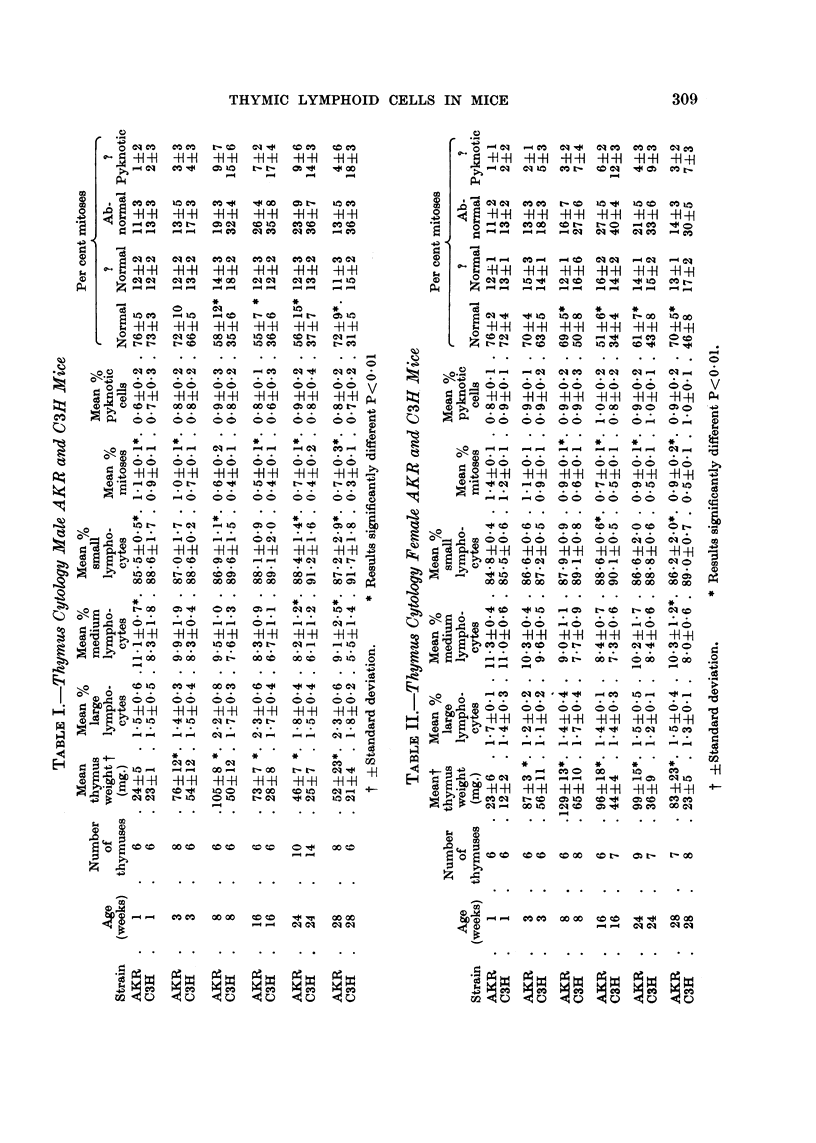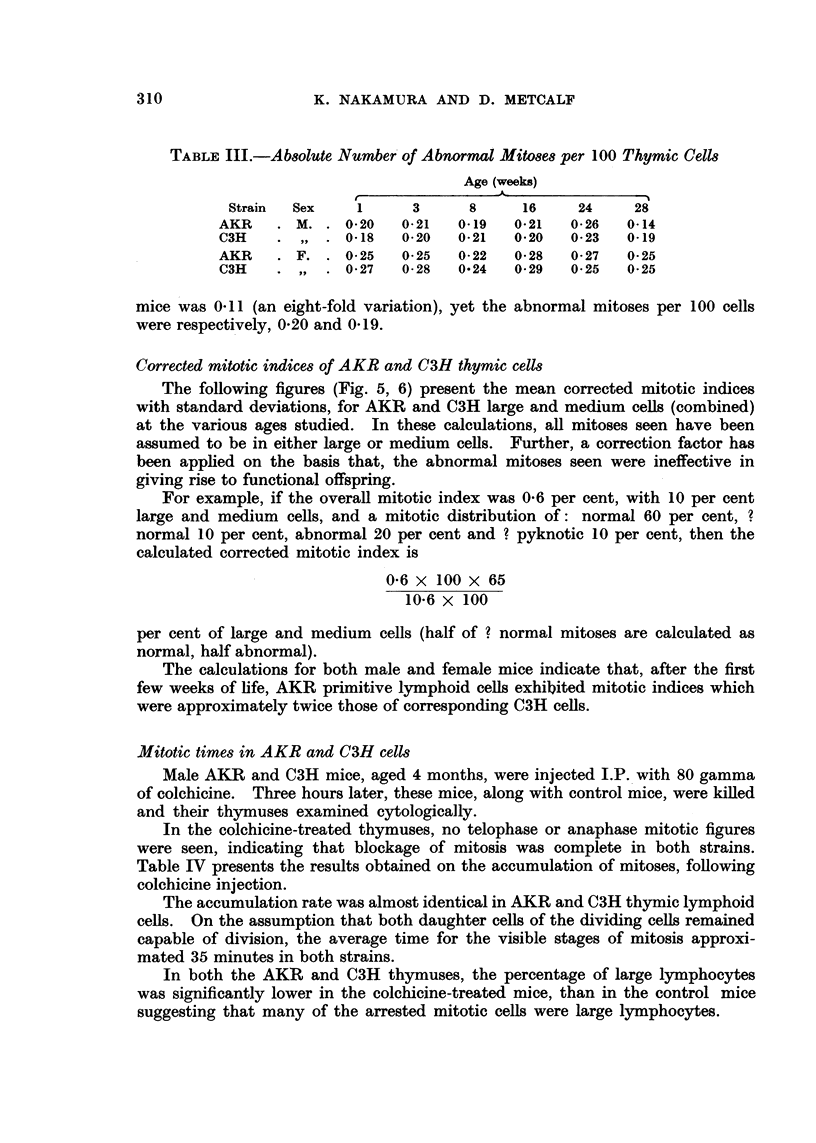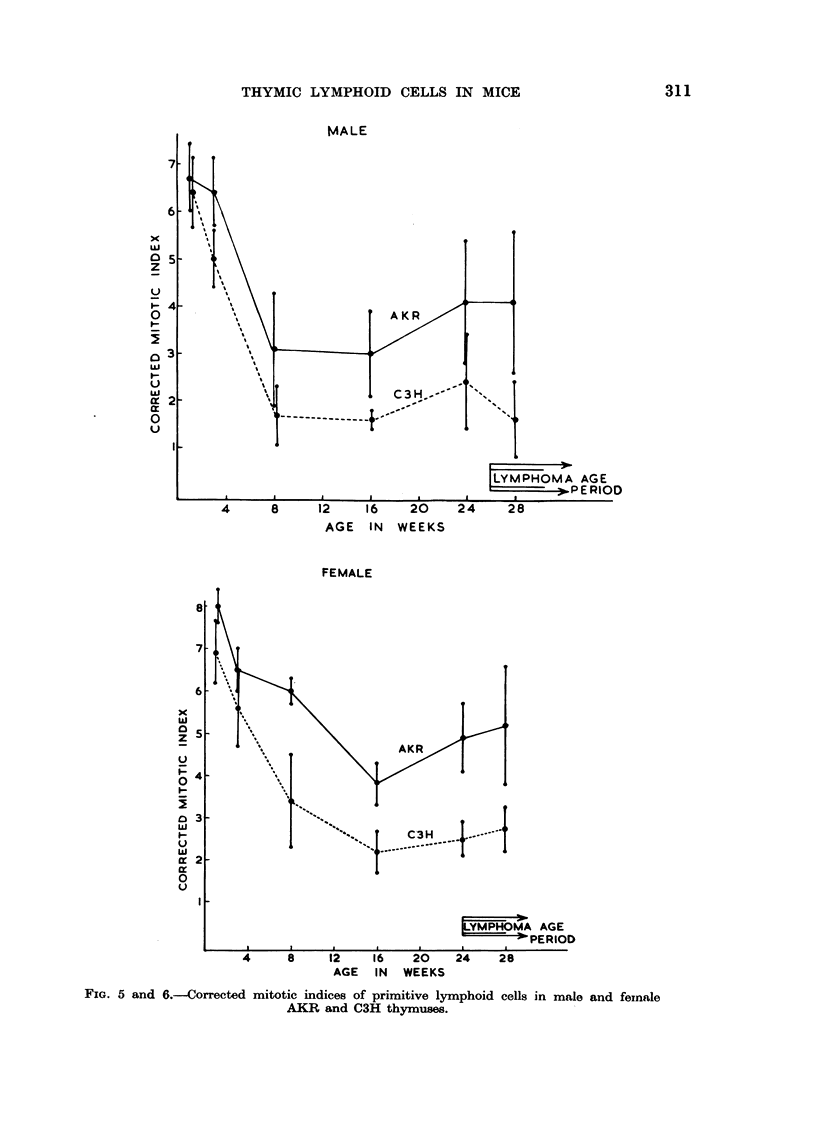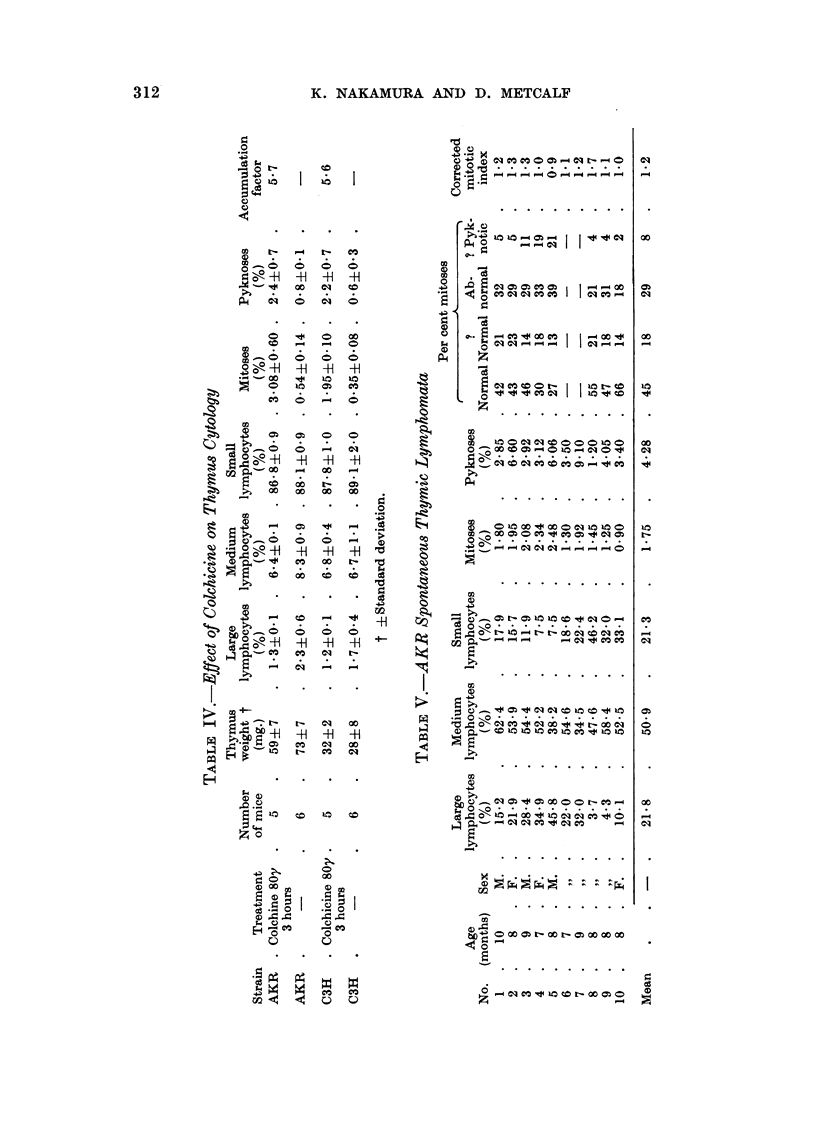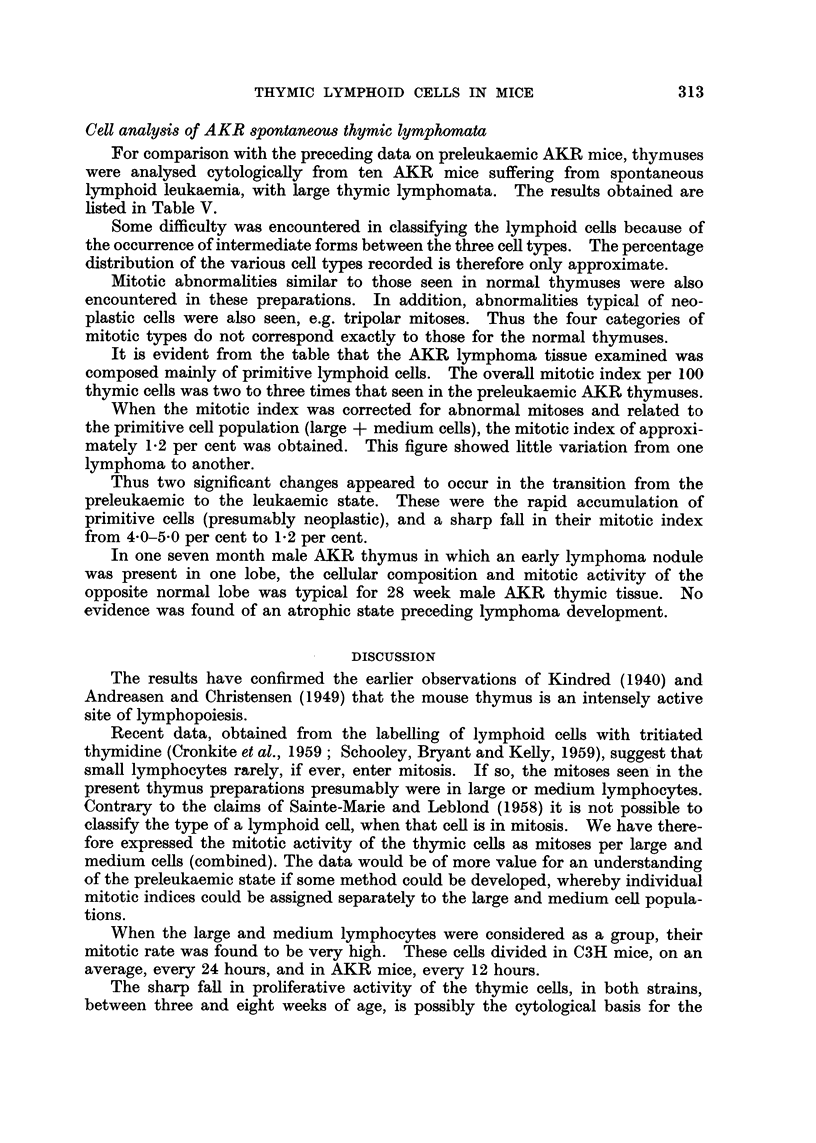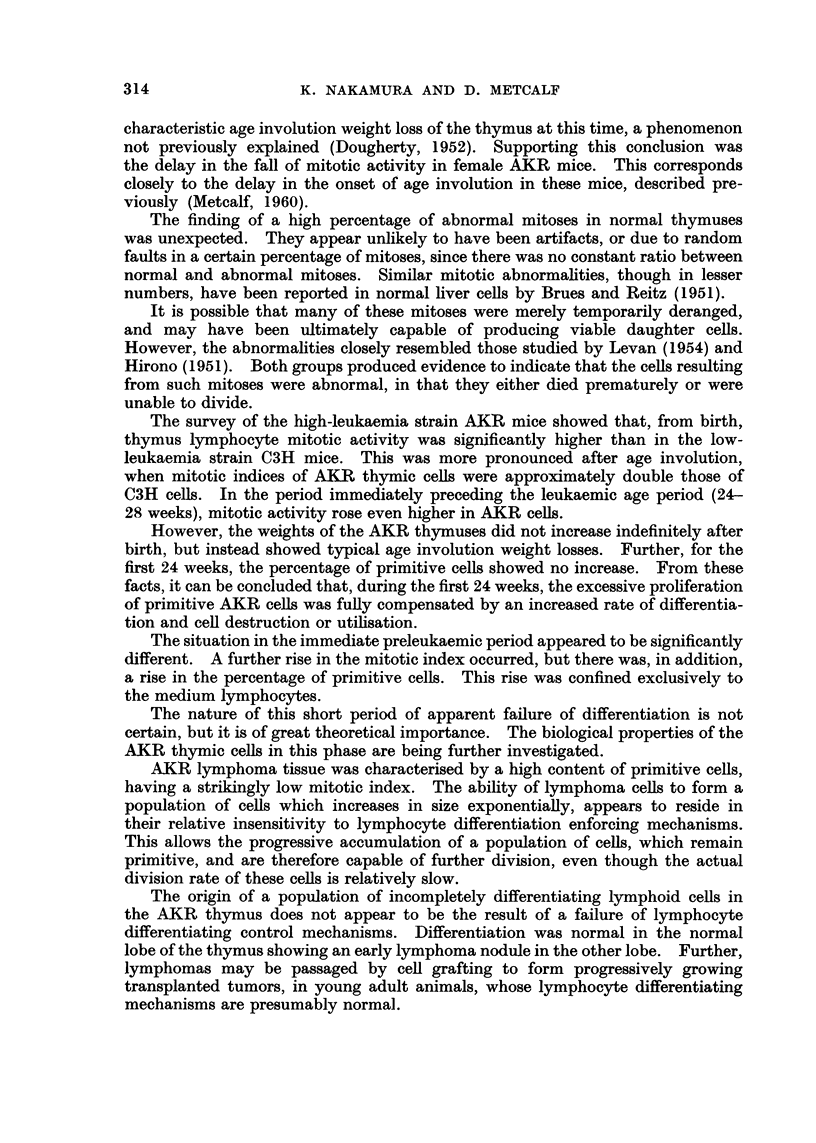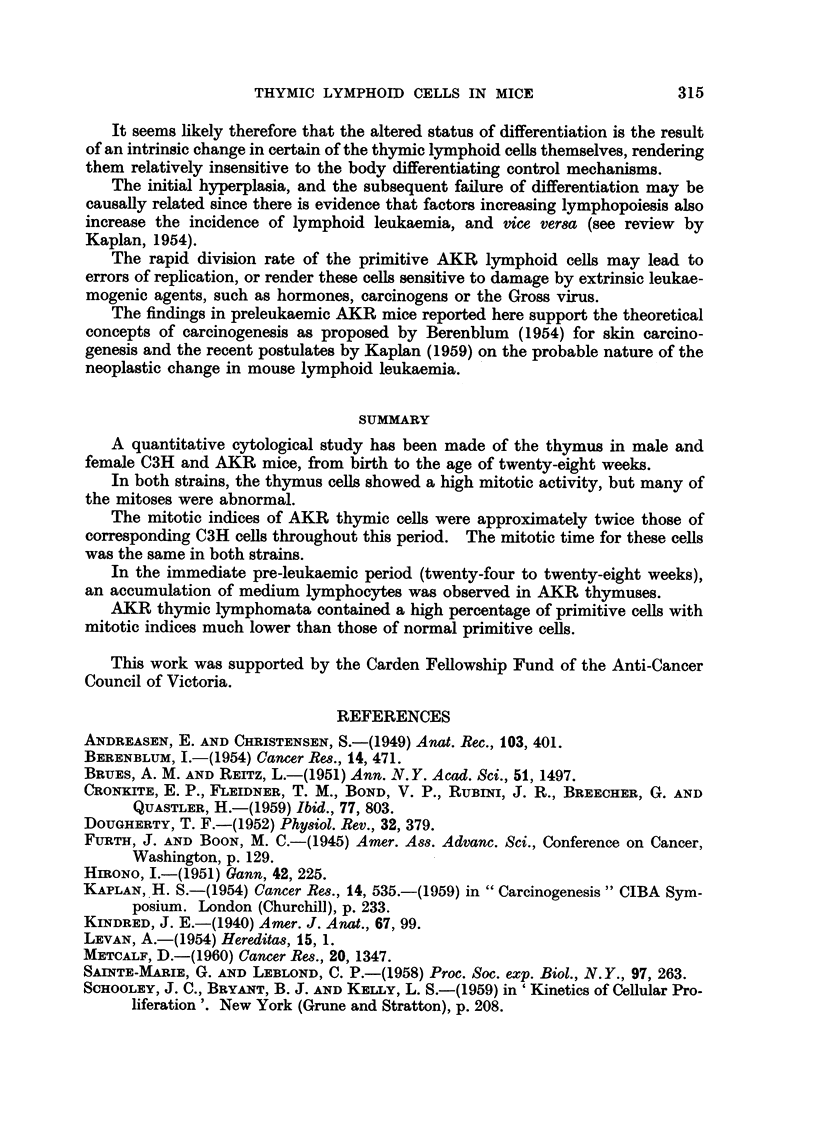# Quantitative Cytological Studies on Thymic Lymphoid Cells in Normal, Preleukaemic and Leukaemic Mice

**DOI:** 10.1038/bjc.1961.38

**Published:** 1961-06

**Authors:** K. Nakamura, D. Metcalf

## Abstract

**Images:**


### QUANTITATIVE CYTOLOGICAL STUDIES ON THYMIC LYMPHOID CELLS IN NORMAL, PRELEUKAEMIC AND LEUKAEMIC MICE

K. NAKAMURA AND D. METCALF

*From the Cancer Research Laboratory, Walter and Eliza Hall Institute, Royal Melbourne Hospital, Melbourne, Victoria, Australia*

Received for publication February 1, 1961

THE incidence of spontaneous thymic lymphomata, with generalised lymphoid leukaemia, in AKR mice varies from 85–95 per cent. The first histological and biological evidence of leukaemia is usually to be found in the thymus, and the disease is commonly limited to the thymus (Furth and Boon, 1945). The first deaths from this disease usually occur in female mice at six months, and in male mice at seven months of age.

The AKR thymus therefore presents an ideal opportunity for a detailed cytological examination of the preleukaemic state. In the sense used in this paper, " preleukaemic " refers merely to AKR mice in the 0–7 months age period.

This paper presents the results of a comparative survey of AKR and C3H thymuses in this age period.

### MATERIALS AND METHODS

### *Mice*

Mice used were inbred males and females of strains AKR and C3H. The AKR mice (current lymphoid leukaemia incidence 90 per cent) were originally obtained from Dr. Jacob Furth. The C3H mice (current lymphoid leukaemia incidence 1–5 per cent) originated in the Imperial Cancer Research Fund Laboratories, London.

The mice were housed in metal tins, with sawdust bedding. The diet consisted of purina chow and water *ad lib.*, with carrots twice weekly and occasional greens. The room temperature of the animal colonies was maintained at 75° F. The AKR and C3H colonies were housed in separate, but adjacent animal rooms.

### *Cytological preparations*

Cytological preparations of thymus cells were always made between 2 and 4 p.m. Mice were killed with ether, and the thymuses removed and weighed. The thymuses were then placed in separate Petri dishes, and 0·25 ml. saline added. The thymuses were finely minced with sharp curved scissors. An equal volume of glacial acetic acid/orcein was added and the mince mashed into minute fragments with angulated forceps. Acetic acid/gentian violet was then added (final concentration, 2 parts acetic acid/orcein : 1 part acetic acid/gentian violet).

Several drops of liquid glycerine were added to the mince, and a drop of the mince was placed on a slide. A cover slip was then gently lowered on the drop and pressed down on the slide until adequate cell spreading was obtained. The preparations were sealed with balsam/paraffin, and stored at 4° C.

*Sampling technique*

In these preparations, segregation of cell types occurred when the cover slip was lowered on the drop. The preparations were therefore sampled in a fixed battlement pattern, passing from one end of the cover slip to the other.

In each preparation, 5000 consecutive lymphocytes and 100 consecutive mitoses were typed.

*Cell typing*

The general appearance of these thymus preparations is shown in Fig. 1 and 2. In these preparations, the size of the lymphoid cells was to a large degree determined by the degree of spreading occurring during their preparation. Accordingly, lymphoid cells were typed on the basis of nuclear detail, little attention being paid to the apparent size of the cells. Fig. 3 shows the nuclear details of the cell types found in these preparations.

The epithelial/reticular cells were very few in number, relative to the number of lymphoid cells, and were not counted in this survey.

*Mitotic classification*

Only cells showing distinct chromosomes were scored as mitoses, cells in early prophase were not included in the mitotic count.

Mitoses classified as ? normal were cells in which the mitotic figure was not clearly visible. Cells in which it was doubtful whether the nucleus showed intense clumping of chromosomes or was merely pyknotic, were classified as ? pyknotic.

*Statistical analysis*

Statistical analyses were made using the Student " *t* " series method.

### RESULTS

Thymuses were examined from male and female AKR mice of various ages—1, 3, 8, 16, 24 and 28 weeks. For comparison, thymuses were examined from male and female C3H mice of the same ages. The thymus weights of the mice examined (Tables I and II) were within the normal limits previously described for these strains (Metcalf, 1960).

*Cell types*

Table I shows the mean percentages of the various lymphoid cell types in male AKR and C3H thymuses of various ages. Significant strain differences were found in the percentages of medium and small lymphocytes.

In C3H mice, the percentage of medium lymphocytes fell progressively with increasing age. In AKR mice, the percentage of this cell type was significantly higher than in C3H mice. After an initial fall between 1 and 24 weeks, the percentage rose in the immediate preleukaemic period (24–28 weeks). Considerable individual variation (from 5·4–12·5 per cent) was encountered in the medium lymphocyte content of 28 week AKR thymuses.

The percentage of small lymphocytes in AKR thymuses was lower than in C3H thymuses, at all ages.

The survey of female AKR and C3H thymuses (Table II) revealed similar strain differences to those described in the male mice. Again there was a rise in the percentage of medium lymphocytes in AKR thymuses at 28 weeks, and there was considerable individual variation (from 8·4–14·8 per cent) in the medium lymphocyte content of 24 and 28 week old AKR thymuses.

*Mitotic indices*

The overall mitotic index (mitoses per 100 thymic cells) was maximal in male and female C3H thymuses at one week (Tables I and II), and fell sharply at the onset of age involution, between three and eight weeks. After this time, the mitotic index remained relatively constant.

The mitotic index of AKR thymic cells, at all ages, was higher than that of corresponding C3H cells. As in C3H thymuses, a fall in the index occurred at age involution. In the female AKR thymuses, this fall was delayed until after eight weeks. After 16 weeks, mitotic indices rose in AKR thymic cells, in both sexes, so that by 28 weeks, the indices averaged twice those of corresponding C3H cells. Again, considerable individual variation was encountered in AKR thymuses in the 28 week age group, values ranging from 0·30–1·12 per cent.

It will be noted that, at all ages, female AKR mitotic indices were consistently higher than corresponding male indices.

*Mitotic abnormalities*

A high frequency of abnormal mitoses was encountered in these thymus preparations. The most common abnormalities, in order of frequency were, (*a*) metaphase arrest, with chromosome swelling and stickiness, (*b*) failure of cytoplasmic division, (*c*) chromosome bridges and (*d*) chromosome clumping (Fig. 4).

The percentage incidence of mitotic abnormalities in AKR and C3H thymuses at various ages is shown in Tables I and II. In C3H thymuses, the percentage frequency of abnormal mitoses rose sharply after age involution, reaching levels of 60 per cent. A similar change was found in AKR thymuses, but the frequency of abnormal mitoses never exceeded 40 per cent, and fell after 24 weeks.

However, calculation of the absolute number of abnormal mitoses per 100 thymic cells (Table III) indicated that there was no strain difference in their incidence, and that there was little fluctuation with age. This was in contrast to the fluctuations in the absolute number of normal mitoses present in these thymuses. For example, in male mice, the number of normal mitoses per 100 cells in one week old AKR mice was 0·90, whilst the number in 28 week old C3H

---

EXPLANATION OF PLATE

FIG. 1.—General view of cell preparation from normal six-months AKR thymus. Note absence of damaged cells. ×220.

FIG. 2.—General view of cell preparation from AKR thymic lymphoma. Note preponderance of primitive cells. ×220.

FIG. 3.—Nuclear details of the various types of lymphoid cells from a normal six month AKR thymus. ×560. L, large lymphocyte. M, medium lymphocyte. S, small lymphocyte.

FIG. 4.—Mitotic abnormalities in normal thymic lymphoid cells. A and B chromosome bridges. C and D Metaphase arrest, with swelling and stickiness of chromosomes. E Failure of cytoplasmic division at telophase. F Binucleate cell. ×1000.

1

2

3

4

Nakamura and Metcalf.

TABLE I.—*Thymus Cytology Male AKR and C3H Mice*

| Strain | Age (weeks) | Number of thymuses | Mean thymus weight † (mg.) | Mean % large lympho-cytes | Mean % medium lympho-cytes | Mean % small lympho-cytes | Mean % mitoses | Mean % pyknotic cells | Per cent mitoses: Normal | ? Normal | Ab-normal | ? Pyknotic |
|---|---|---|---|---|---|---|---|---|---|---|---|---|
| AKR | 1 | 6 | 24±5 | 1·5±0·6 | 11·1±0·7* | 85·5±0·5* | 1·1±0·1* | 0·6±0·2 | 76±5 | 12±2 | 11±3 | 1±2 |
| C3H | 1 | 6 | 23±1 | 1·5±0·5 | 8·3±1·8 | 88·6±1·7 | 0·9±0·1 | 0·7±0·3 | 73±3 | 12±2 | 13±3 | 2±3 |
| AKR | 3 | 8 | 76±12* | 1·4±0·3 | 9·9±1·9 | 87·0±1·7 | 1·0±0·1* | 0·8±0·2 | 72±10 | 12±2 | 13±5 | 3±3 |
| C3H | 3 | 6 | 54±12 | 1·5±0·4 | 8·3±0·4 | 88·6±0·2 | 0·7±0·1 | 0·8±0·2 | 66±5 | 13±2 | 17±3 | 4±3 |
| AKR | 8 | 6 | 105±8 * | 2·2±0·8 | 9·5±1·0 | 86·9±1·1* | 0·6±0·2 | 0·9±0·3 | 58±12* | 14±3 | 19±3 | 9±7 |
| C3H | 8 | 6 | 50±12 | 1·7±0·3 | 7·6±1·3 | 89·6±1·5 | 0·4±0·1 | 0·8±0·2 | 35±6 | 18±2 | 32±4 | 15±6 |
| AKR | 16 | 6 | 73±7 * | 2·3±0·6 | 8·3±0·9 | 88·1±0·9 | 0·5±0·1* | 0·8±0·1 | 55±7 * | 12±3 | 26±4 | 7±2 |
| C3H | 16 | 6 | 28±8 | 1·7±0·4 | 6·7±1·1 | 89·1±2·0 | 0·4±0·1 | 0·6±0·3 | 36±6 | 12±2 | 35±8 | 17±4 |
| AKR | 24 | 10 | 46±7 * | 1·8±0·4 | 8·2±1·2* | 88·4±1·4* | 0·7±0·1* | 0·9±0·2 | 56±15* | 12±3 | 23±9 | 9±6 |
| C3H | 24 | 14 | 25±7 | 1·5±0·4 | 6·1±1·2 | 91·2±1·6 | 0·4±0·2 | 0·8±0·4 | 37±7 | 13±2 | 36±7 | 14±3 |
| AKR | 28 | 8 | 52±23* | 2·3±0·6 | 9·1±2·5* | 87·2±2·9* | 0·7±0·3* | 0·8±0·2 | 72±9* | 11±3 | 13±5 | 4±6 |
| C3H | 28 | 6 | 21±4 | 1·8±0·2 | 5·5±1·4 | 91·7±1·8 | 0·3±0·1 | 0·7±0·2 | 31±5 | 15±2 | 36±3 | 18±3 |

† ±Standard deviation. * Results significantly different $P<0\cdot01$

TABLE II.—*Thymus Cytology Female AKR and C3H Mice*

| Strain | Age (weeks) | Number of thymuses | Mean† thymus weight (mg.) | Mean % large lympho-cytes | Mean % medium lympho-cytes | Mean % small lympho-cytes | Mean % mitoses | Mean % pyknotic cells | Per cent mitoses: Normal | ? Normal | Ab-normal | ? Pyknotic |
|---|---|---|---|---|---|---|---|---|---|---|---|---|
| AKR | 1 | 6 | 23±6 | 1·7±0·1 | 11·3±0·4 | 84·8±0·4 | 1·4±0·1 | 0·8±0·1 | 76±2 | 12±1 | 11±2 | 1±1 |
| C3H | 1 | 6 | 12±2 | 1·4±0·3 | 11·0±0·6 | 85·5±0·6 | 1·2±0·1 | 0·9±0·1 | 72±4 | 13±1 | 13±2 | 2±2 |
| AKR | 3 | 6 | 87±3 * | 1·2±0·2 | 10·3±0·4 | 86·6±0·6 | 1·1±0·1 | 0·9±0·1 | 70±4 | 15±3 | 13±3 | 2±1 |
| C3H | 3 | 6 | 56±11 | 1·1±0·2 | 9·6±0·5 | 87·2±0·5 | 0·9±0·1 | 0·9±0·2 | 63±5 | 14±1 | 18±3 | 5±3 |
| AKR | 8 | 6 | 129±13* | 1·4±0·4 | 9·0±1·1 | 87·9±0·9 | 0·9±0·1* | 0·9±0·2 | 69±5* | 12±1 | 16±7 | 3±2 |
| C3H | 8 | 8 | 65±10 | 1·7±0·4 | 7·7±0·9 | 89·1±0·8 | 0·6±0·1 | 0·9±0·3 | 50±8 | 16±6 | 27±6 | 7±4 |
| AKR | 16 | 6 | 96±18* | 1·4±0·1 | 8·4±0·7 | 88·6±0·6* | 0·7±0·1* | 1·0±0·2 | 51±6* | 16±2 | 27±5 | 6±2 |
| C3H | 16 | 7 | 44±4 | 1·4±0·3 | 7·3±0·6 | 90·1±0·5 | 0·5±0·1 | 0·8±0·2 | 34±4 | 14±2 | 40±4 | 12±3 |
| AKR | 24 | 9 | 99±15* | 1·5±0·5 | 10·2±1·7 | 86·6±2·0 | 0·9±0·1* | 0·9±0·2 | 61±7* | 14±1 | 21±5 | 4±3 |
| C3H | 24 | 7 | 36±9 | 1·2±0·1 | 8·4±0·6 | 88·8±0·6 | 0·5±0·1 | 1·0±0·1 | 43±8 | 15±2 | 33±6 | 9±3 |
| AKR | 28 | 7 | 83±23* | 1·5±0·4 | 10·3±1·2* | 86·2±2·0* | 0·9±0·2* | 0·9±0·2 | 70±5* | 13±1 | 14±3 | 3±2 |
| C3H | 28 | 8 | 23±5 | 1·3±0·1 | 8·0±0·6 | 89·0±0·7 | 0·5±0·1 | 1·0±0·1 | 46±8 | 17±2 | 30±5 | 7±3 |

† ±Standard deviation. * Results significantly different $P<0\cdot01$.

TABLE III.—*Absolute Number of Abnormal Mitoses per* 100 *Thymic Cells*

| Strain | Sex | Age (weeks) 1 | 3 | 8 | 16 | 24 | 28 |
|---|---|---|---|---|---|---|---|
| AKR | M. | 0·20 | 0·21 | 0·19 | 0·21 | 0·26 | 0·14 |
| C3H | „ | 0·18 | 0·20 | 0·21 | 0·20 | 0·23 | 0·19 |
| AKR | F. | 0·25 | 0·25 | 0·22 | 0·28 | 0·27 | 0·25 |
| C3H | „ | 0·27 | 0·28 | 0·24 | 0·29 | 0·25 | 0·25 |

mice was 0·11 (an eight-fold variation), yet the abnormal mitoses per 100 cells were respectively, 0·20 and 0·19.

*Corrected mitotic indices of AKR and C3H thymic cells*

The following figures (Fig. 5, 6) present the mean corrected mitotic indices with standard deviations, for AKR and C3H large and medium cells (combined) at the various ages studied. In these calculations, all mitoses seen have been assumed to be in either large or medium cells. Further, a correction factor has been applied on the basis that, the abnormal mitoses seen were ineffective in giving rise to functional offspring.

For example, if the overall mitotic index was 0·6 per cent, with 10 per cent large and medium cells, and a mitotic distribution of: normal 60 per cent, ? normal 10 per cent, abnormal 20 per cent and ? pyknotic 10 per cent, then the calculated corrected mitotic index is

$$\frac{0{\cdot}6 \times 100 \times 65}{10{\cdot}6 \times 100}$$

per cent of large and medium cells (half of ? normal mitoses are calculated as normal, half abnormal).

The calculations for both male and female mice indicate that, after the first few weeks of life, AKR primitive lymphoid cells exhibited mitotic indices which were approximately twice those of corresponding C3H cells.

*Mitotic times in AKR and C3H cells*

Male AKR and C3H mice, aged 4 months, were injected I.P. with 80 gamma of colchicine. Three hours later, these mice, along with control mice, were killed and their thymuses examined cytologically.

In the colchicine-treated thymuses, no telophase or anaphase mitotic figures were seen, indicating that blockage of mitosis was complete in both strains. Table IV presents the results obtained on the accumulation of mitoses, following colchicine injection.

The accumulation rate was almost identical in AKR and C3H thymic lymphoid cells. On the assumption that both daughter cells of the dividing cells remained capable of division, the average time for the visible stages of mitosis approximated 35 minutes in both strains.

In both the AKR and C3H thymuses, the percentage of large lymphocytes was significantly lower in the colchicine-treated mice, than in the control mice suggesting that many of the arrested mitotic cells were large lymphocytes.

FIG. 5 and 6.—Corrected mitotic indices of primitive lymphoid cells in male and female AKR and C3H thymuses.

TABLE IV.—*Effect of Colchicine on Thymus Cytology*

| Strain | Treatment | Number of mice | Thymus weight † (mg.) | Large lymphocytes (%) | Medium lymphocytes (%) | Small lymphocytes (%) | Mitoses (%) | Pyknoses (%) | Accumulation factor |
|---|---|---|---|---|---|---|---|---|---|
| AKR | Colchine 80γ 3 hours | 5 | 59±7 | 1·3±0·1 | 6·4±0·1 | 86·8±0·9 | 3·08±0·60 | 2·4±0·7 | 5·7 |
| AKR | — | 6 | 73±7 | 2·3±0·6 | 8·3±0·9 | 88·1±0·9 | 0·54±0·14 | 0·8±0·1 | — |
| C3H | Colchicine 80γ 3 hours | 5 | 32±2 | 1·2±0·1 | 6·8±0·4 | 87·8±1·0 | 1·95±0·10 | 2·2±0·7 | 5·6 |
| C3H | — | 6 | 28±8 | 1·7±0·4 | 6·7±1·1 | 89·1±2·0 | 0·35±0·08 | 0·6±0·3 | — |

† ±Standard deviation.

TABLE V.—*AKR Spontaneous Thymic Lymphomata*

| No. | Age (months) | Sex | Large lymphocytes (%) | Medium lymphocytes (%) | Small lymphocytes (%) | Mitoses (%) | Pyknoses (%) | Per cent mitoses: Normal | ? Normal | Ab-normal | ? Pyk-notic | Corrected mitotic index |
|---|---|---|---|---|---|---|---|---|---|---|---|---|
| 1 | 10 | M. | 15·2 | 62·4 | 17·9 | 1·80 | 2·85 | 42 | 21 | 32 | 5 | 1·2 |
| 2 | 8 | F. | 21·9 | 53·9 | 15·7 | 1·95 | 6·60 | 43 | 23 | 29 | 5 | 1·3 |
| 3 | 9 | M. | 28·4 | 54·4 | 11·9 | 2·08 | 2·92 | 46 | 14 | 29 | 11 | 1·3 |
| 4 | 7 | F. | 34·9 | 52·2 | 7·5 | 2·34 | 3·12 | 30 | 18 | 33 | 19 | 1·0 |
| 5 | 8 | M. | 45·8 | 38·2 | 7·5 | 2·48 | 6·06 | 27 | 13 | 39 | 21 | 0·9 |
| 6 | 7 | ,, | 22·0 | 54·6 | 18·6 | 1·30 | 3·50 | — | — | — | — | 1·1 |
| 7 | 9 | ,, | 32·0 | 34·5 | 22·4 | 1·92 | 9·10 | — | — | — | — | 1·2 |
| 8 | 8 | ,, | 3·7 | 47·6 | 46·2 | 1·45 | 1·20 | 55 | 21 | 21 | 4 | 1·7 |
| 9 | 8 | ,, | 4·3 | 58·4 | 32·0 | 1·25 | 4·05 | 47 | 18 | 31 | 4 | 1·1 |
| 10 | 8 | F. | 10·1 | 52·5 | 33·1 | 0·90 | 3·40 | 66 | 14 | 18 | 2 | 1·0 |
| Mean | | — | 21·8 | 50·9 | 21·3 | 1·75 | 4·28 | 45 | 18 | 29 | 8 | 1·2 |

*Cell analysis of AKR spontaneous thymic lymphomata*

For comparison with the preceding data on preleukaemic AKR mice, thymuses were analysed cytologically from ten AKR mice suffering from spontaneous lymphoid leukaemia, with large thymic lymphomata. The results obtained are listed in Table V.

Some difficulty was encountered in classifying the lymphoid cells because of the occurrence of intermediate forms between the three cell types. The percentage distribution of the various cell types recorded is therefore only approximate.

Mitotic abnormalities similar to those seen in normal thymuses were also encountered in these preparations. In addition, abnormalities typical of neoplastic cells were also seen, e.g. tripolar mitoses. Thus the four categories of mitotic types do not correspond exactly to those for the normal thymuses.

It is evident from the table that the AKR lymphoma tissue examined was composed mainly of primitive lymphoid cells. The overall mitotic index per 100 thymic cells was two to three times that seen in the preleukaemic AKR thymuses.

When the mitotic index was corrected for abnormal mitoses and related to the primitive cell population (large + medium cells), the mitotic index of approximately 1·2 per cent was obtained. This figure showed little variation from one lymphoma to another.

Thus two significant changes appeared to occur in the transition from the preleukaemic to the leukaemic state. These were the rapid accumulation of primitive cells (presumably neoplastic), and a sharp fall in their mitotic index from 4·0–5·0 per cent to 1·2 per cent.

In one seven month male AKR thymus in which an early lymphoma nodule was present in one lobe, the cellular composition and mitotic activity of the opposite normal lobe was typical for 28 week male AKR thymic tissue. No evidence was found of an atrophic state preceding lymphoma development.

### DISCUSSION

The results have confirmed the earlier observations of Kindred (1940) and Andreasen and Christensen (1949) that the mouse thymus is an intensely active site of lymphopoiesis.

Recent data, obtained from the labelling of lymphoid cells with tritiated thymidine (Cronkite *et al.*, 1959 ; Schooley, Bryant and Kelly, 1959), suggest that small lymphocytes rarely, if ever, enter mitosis. If so, the mitoses seen in the present thymus preparations presumably were in large or medium lymphocytes. Contrary to the claims of Sainte-Marie and Leblond (1958) it is not possible to classify the type of a lymphoid cell, when that cell is in mitosis. We have therefore expressed the mitotic activity of the thymic cells as mitoses per large and medium cells (combined). The data would be of more value for an understanding of the preleukaemic state if some method could be developed, whereby individual mitotic indices could be assigned separately to the large and medium cell populations.

When the large and medium lymphocytes were considered as a group, their mitotic rate was found to be very high. These cells divided in C3H mice, on an average, every 24 hours, and in AKR mice, every 12 hours.

The sharp fall in proliferative activity of the thymic cells, in both strains, between three and eight weeks of age, is possibly the cytological basis for the characteristic age involution weight loss of the thymus at this time, a phenomenon not previously explained (Dougherty, 1952). Supporting this conclusion was the delay in the fall of mitotic activity in female AKR mice. This corresponds closely to the delay in the onset of age involution in these mice, described previously (Metcalf, 1960).

The finding of a high percentage of abnormal mitoses in normal thymuses was unexpected. They appear unlikely to have been artifacts, or due to random faults in a certain percentage of mitoses, since there was no constant ratio between normal and abnormal mitoses. Similar mitotic abnormalities, though in lesser numbers, have been reported in normal liver cells by Brues and Reitz (1951).

It is possible that many of these mitoses were merely temporarily deranged, and may have been ultimately capable of producing viable daughter cells. However, the abnormalities closely resembled those studied by Levan (1954) and Hirono (1951). Both groups produced evidence to indicate that the cells resulting from such mitoses were abnormal, in that they either died prematurely or were unable to divide.

The survey of the high-leukaemia strain AKR mice showed that, from birth, thymus lymphocyte mitotic activity was significantly higher than in the low-leukaemia strain C3H mice. This was more pronounced after age involution, when mitotic indices of AKR thymic cells were approximately double those of C3H cells. In the period immediately preceding the leukaemic age period (24–28 weeks), mitotic activity rose even higher in AKR cells.

However, the weights of the AKR thymuses did not increase indefinitely after birth, but instead showed typical age involution weight losses. Further, for the first 24 weeks, the percentage of primitive cells showed no increase. From these facts, it can be concluded that, during the first 24 weeks, the excessive proliferation of primitive AKR cells was fully compensated by an increased rate of differentiation and cell destruction or utilisation.

The situation in the immediate preleukaemic period appeared to be significantly different. A further rise in the mitotic index occurred, but there was, in addition, a rise in the percentage of primitive cells. This rise was confined exclusively to the medium lymphocytes.

The nature of this short period of apparent failure of differentiation is not certain, but it is of great theoretical importance. The biological properties of the AKR thymic cells in this phase are being further investigated.

AKR lymphoma tissue was characterised by a high content of primitive cells, having a strikingly low mitotic index. The ability of lymphoma cells to form a population of cells which increases in size exponentially, appears to reside in their relative insensitivity to lymphocyte differentiation enforcing mechanisms. This allows the progressive accumulation of a population of cells, which remain primitive, and are therefore capable of further division, even though the actual division rate of these cells is relatively slow.

The origin of a population of incompletely differentiating lymphoid cells in the AKR thymus does not appear to be the result of a failure of lymphocyte differentiating control mechanisms. Differentiation was normal in the normal lobe of the thymus showing an early lymphoma nodule in the other lobe. Further, lymphomas may be passaged by cell grafting to form progressively growing transplanted tumors, in young adult animals, whose lymphocyte differentiating mechanisms are presumably normal.

It seems likely therefore that the altered status of differentiation is the result of an intrinsic change in certain of the thymic lymphoid cells themselves, rendering them relatively insensitive to the body differentiating control mechanisms.

The initial hyperplasia, and the subsequent failure of differentiation may be causally related since there is evidence that factors increasing lymphopoiesis also increase the incidence of lymphoid leukaemia, and *vice versa* (see review by Kaplan, 1954).

The rapid division rate of the primitive AKR lymphoid cells may lead to errors of replication, or render these cells sensitive to damage by extrinsic leukaemogenic agents, such as hormones, carcinogens or the Gross virus.

The findings in preleukaemic AKR mice reported here support the theoretical concepts of carcinogenesis as proposed by Berenblum (1954) for skin carcinogenesis and the recent postulates by Kaplan (1959) on the probable nature of the neoplastic change in mouse lymphoid leukaemia.

### SUMMARY

A quantitative cytological study has been made of the thymus in male and female C3H and AKR mice, from birth to the age of twenty-eight weeks.

In both strains, the thymus cells showed a high mitotic activity, but many of the mitoses were abnormal.

The mitotic indices of AKR thymic cells were approximately twice those of corresponding C3H cells throughout this period. The mitotic time for these cells was the same in both strains.

In the immediate pre-leukaemic period (twenty-four to twenty-eight weeks), an accumulation of medium lymphocytes was observed in AKR thymuses.

AKR thymic lymphomata contained a high percentage of primitive cells with mitotic indices much lower than those of normal primitive cells.

This work was supported by the Carden Fellowship Fund of the Anti-Cancer Council of Victoria.

### REFERENCES

Andreasen, E. and Christensen, S.—(1949) *Anat. Rec.*, **103**, 401.
Berenblum, I.—(1954) *Cancer Res.*, **14**, 471.
Brues, A. M. and Reitz, L.—(1951) *Ann. N.Y. Acad. Sci.*, **51**, 1497.
Cronkite, E. P., Fleidner, T. M., Bond, V. P., Rubini, J. R., Breecher, G. and Quastler, H.—(1959) *Ibid.*, **77**, 803.
Dougherty, T. F.—(1952) *Physiol. Rev.*, **32**, 379.
Furth, J. and Boon, M. C.—(1945) *Amer. Ass. Advanc. Sci.*, Conference on Cancer, Washington, p. 129.
Hirono, I.—(1951) *Gann*, **42**, 225.
Kaplan, H. S.—(1954) *Cancer Res.*, **14**, 535.—(1959) in " Carcinogenesis " CIBA Symposium. London (Churchill), p. 233.
Kindred, J. E.—(1940) *Amer. J. Anat.*, **67**, 99.
Levan, A.—(1954) *Hereditas*, **15**, 1.
Metcalf, D.—(1960) *Cancer Res.*, **20**, 1347.
Sainte-Marie, G. and Leblond, C. P.—(1958) *Proc. Soc. exp. Biol., N.Y.*, **97**, 263.
Schooley, J. C., Bryant, B. J. and Kelly, L. S.—(1959) in ' Kinetics of Cellular Proliferation '. New York (Grune and Stratton), p. 208.